# Abortive Treatment of Extrauterine Fœtation by Means of Electricity

**Published:** 1857-12

**Authors:** 

**Affiliations:** Pisa


### ABORTIVE TREATMENT OF EXTRAUTERINE FŒTATION BY MEANS OF ELECTRICITY.

BY DR. BURCI OF PISA.

A young woman, aged 29 years, who had borne four children, on the 1st of July, 1852, experienced symptoms of her fifth pregnancy as she supposed. She remained ordinarily well until the 29th of December, when she was suddenly seized with pain in the hypogastric region, bearing down, dysuria and tenesmus, and fainted several times. As she feared abortion, she sent for a physician. Examination revealed a natural condition of the uterine cervix. The symptoms were met by cataplasms, belladonna ointment and castor oil. They continued, however, for some time, returning often after partial relief, until a considerable quantity of blood with albuminous floculi were discharged from the uterus. On the 25th of January, 1853, a tumor about the size of an orange was discovered occupying the left iliac fossa, which the author and several of his colleagues diagnosticated tubal fœtation. The indication seemed to be to arrest the growth of the ovum. To effect this, two needles were introduced, one on each side of the tumor, with their points toward its center. Their remote ends were connected with the opposite poles of a galvanic battery, and a stream of electricity thus directed through the middle of the swelling. The patient screamed with pain, which this procedure occasioned. From this forward the tumor diminished in size. On the 6th of March it was only about as large as a pigeon's egg, and in April the menses returned. No further treatment was necessary.—*L' Union Med.*

